# A Multimodal Dense Parallel Global Attention Mechanism for Brain Tumor Image Segmentation

**DOI:** 10.3390/jimaging12060255

**Published:** 2026-06-09

**Authors:** Zhuye Xu, Ru Qiao

**Affiliations:** School of New Energy and Power Engineering, Lanzhou Jiaotong University, Lanzhou 730070, China; xuzhuye@mail.lzjtu.cn

**Keywords:** brain tumor, feature recombination, image segmentation, multimodal

## Abstract

Brain tumor segmentation from 3D MRI presents significant challenges due to small lesion sizes, ambiguous boundaries, arbitrary spatial distributions, and heterogeneous morphological properties. To tackle these issues, this paper presents a fully automatic 3D brain tumor segmentation network that integrates morphological and anatomical information under a multi-task learning framework for whole tumor, tumor core, and enhanced tumor segmentation. We propose a multimodal feature fusion module to adaptively weight features from four MRI modalities (T1, T1ce, T2, FLAIR), enabling discriminative information integration and helping reduce modality intensity discrepancy and data imbalance. Furthermore, a ConvReXt downsampling module is introduced to preserve fine-grained semantic details by reducing information loss caused by conventional pooling. A dense parallel global attention module is also developed to capture both local details and long-range dependencies, addressing the limited receptive field of standard convolutions. Extensive experiments on the BraTS2020 dataset show that the proposed model obtains average Dice coefficients of 92.54%, 89.21%, and 86.54% for whole tumors, tumor cores, and enhanced tumors. The proposed model achieves competitive performance compared with state-of-the-art methods including nnFormer, validating that it can effectively fuse multimodal and multi-scale features and improve brain tumor segmentation accuracy.

## 1. Introduction

Brain tumors are severe neurological diseases that considerably impair patients’ quality of life and may even be life-threatening. In medical image processing, brain tumor segmentation plays a critical role. Its core objective is to accurately locate and delineate tumor regions from brain images, thereby supporting clinical diagnosis, preoperative planning, treatment strategy design, and longitudinal disease monitoring. Benefiting from the remarkable success of deep learning in image classification, object detection, natural language processing, and other fields, automated brain tumor diagnosis has entered a new era with improved accuracy and efficiency.

In recent years, convolutional neural networks (CNNs) have been widely adopted in medical image processing and have achieved substantial progress [1]. Chen et al. [2] developed the DeepLab series, which strengthened multi-scale feature extraction by using dilated convolutions and spatial pyramid pooling, yielding superior performance in segmenting objects with varying sizes. Zhao et al. [3] presented PSPNet, which formulates image segmentation as multi-scale feature fusion via a pyramid pooling module, thus enhancing the capability to capture both global and local information. Ronneberger et al. [4] introduced U-Net, an encoder–decoder architecture. The encoder extracts features and encodes rich contextual information, while the decoder gradually restores spatial resolution for accurate pixel-wise prediction. With its simple structure and high efficiency, U-Net is especially suitable for medical image segmentation with limited datasets and has become a fundamental backbone, inspiring numerous advanced variants [5]. Zhang et al. [6] proposed Attention-Gated Residual Network (AGResUNet), which suppresses noisy features and enhances tumor-related representations using an attention mechanism. Jiang et al. [7] designed a two-stage cascaded U-Net for brain tumor segmentation: a coarse segmentation is generated in the first stage, and the result is refined in the second stage by fusing the coarse mask with the original image. Although 3D-UNet extracts strong local details from MRI volumes using convolution and pooling, its limited receptive field cannot capture prevalent non-local self-similarities. In addition, constrained by kernel size, single-layer convolution only extracts single-scale features [8].

The encoder–decoder-based U-Net [9] has promoted the advancement of fully automatic brain tumor segmentation. Many improved variants have been proposed, including Swin-unet [10], DenseUNet [11], and VNet [12] for volumetric segmentation. Dong et al. [13] first applied U-Net to brain tumor segmentation. However, their method relies on a 2D U-Net that cannot directly process 3D MRI volumes and must process slices independently. Since 2D U-Net ignores inter-slice contextual information, its segmentation accuracy is restricted. To overcome this limitation, Beers et al. [14] proposed 3D-UNet, which supports direct end-to-end learning on full 3D MRI volumes or sub-volumes. 3D convolution kernels capture both in-plane features and cross-slice dependencies, greatly improving segmentation accuracy. Song et al. [15] introduced a cross-modal attention mechanism and contrastive pre-training to enhance registration by explicitly modeling spatial correspondences across modalities. Nevertheless, deformable registration often sacrifices deformation field smoothness, and performance on real clinical data degrades relative to synthetic data. Although these methods excel at local feature extraction, they still lack the ability to effectively model long-range dependencies and multi-scale contextual information.

Despite the progress achieved by existing brain tumor segmentation methods, several limitations remain. First, brain MRI scans typically include multiple complementary modalities, each emphasizing distinct tissue characteristics. In clinical practice, radiologists rely on different modalities when identifying different tumor subregions, implying that modalities contribute unequally to tumor segmentation. However, most deep learning segmentation methods simply concatenate multi-modal inputs at the image level and assign equal weights to each modality, leaving the complementary information of multi-modal data underexplored. Second, brain tumors—especially enhancing tumor (ET) regions—occupy only a small fraction of the brain volume, leading to severe class imbalance. During training, models tend to be biased toward the dominant background class, reducing the accuracy of small-target lesion detection. Third, while convolutional layers excel at local feature extraction and detail preservation, they are weak at modeling long-range dependencies within images [16]. To overcome these theoretical bottlenecks, drawing insights from robust feature learning paradigms in broader computer vision tasks—such as establishing resilient representations when scaling from simple to complex scenes [17] or utilizing proactive information enhancement to bolster latent feature distinctiveness [18]—has become highly desirable. Building upon existing methodologies, this study proposes a multi-task segmentation network model for 3D brain tumor MRI.

Building upon existing methodologies, this study proposes a multi-task segmentation network model for 3D brain tumor MRI. This model integrates a multimodal feature recombination module and a dense parallel global attention mechanism. The model formulates the segmentation of three distinct tumor regions as independent sub-tasks. The boundaries of the original contributions in this study are explicitly delineated as follows:

1. Development of a Multimodal Feature Recombination Module (MRM) for modality-specific fusion. In contrast to conventional channel attention mechanisms that perform indiscriminate feature recalibration, the core contribution of this module lies in establishing an explicit mapping relationship between modality dimensions and attention weights. It adaptively allocates weights to features of specific physical imaging modalities (e.g., T1, T2, FLAIR, and T1ce). By extracting the physical sensitivity of different modalities to specific brain tumor sub-regions (termed “modality-region specificity”), it replaces generalized feature fusion with targeted lesion signal amplification.

2. Design of a Dense Parallel Global Attention (DGA) module for the synergistic extraction of local and global features. Addressing the gridding artifacts and spatial feature sparsity issues inherent in the existing VAN-LKA architecture—which arise from its reliance on a single dilated convolution—this module explicitly defines the boundary of our algorithmic improvement. Built upon the 3D-UNet framework, the DGA module constructs a dual-parallel feature extraction architecture: the global spatial attention branch discards the single-dilation pathway; while utilizing dilated convolutions to capture long-range dependencies, it integrates a parallel standard convolution to bridge the semantic gaps caused by dilated sampling. Concurrently, the dense block branch is specifically designed to reinforce the extraction and cross-layer reuse of localized detail features. This parallel design circumvents the high computational costs associated with large convolutional kernels while effectively addressing the loss of fine-grained local structures inherent in baseline attention mechanisms.

## 2. Method

### 2.1. A. Overall Structure

The proposed 3D fully automatic brain tumor segmentation network is built upon the 3D U-Net architecture with a multi-branch design. The network mainly comprises an encoder, a decoder, and skip connections, with the goal of improving segmentation accuracy and efficiency. First, the proposed Multimodal Recombination Module (MRM) adaptively learns the weights of different modalities and effectively fuses their complementary features. This facilitates better information integration at each segmentation stage and enables accurate segmentation of different tumor subregions. The recombined feature maps are then fed into the enhanced encoder, which consists of stacked ConvReXt modules, a Dense Global Attention (DGA) module, and a downsampling module to generate the final segmentation output. To present the complete pipeline of the proposed model more clearly, the corresponding pseudocode is given in Algorithm 1:
**Algorithm 1.** Pseudocode for brain tumor segmentation based on multimodal MRI. ReU-Net-Multimodal Brain Tumor SegmentationInput: Multimodal 3D MRI scans (M_t1, M_t1ce, M_t2, M_flair) Output: Segmentation maps (S_wt, S_tc, S_et) Procedure ReU_Net_Segmentation(M_t1, M_t1ce, M_t2, M_flair): 1. Multimodal Feature Recombination F_recombined = Multimodal_Feature_Recombination_Module(M_t1, M_t1ce, M_t2, M_flair) 2. Encoder Path (3D U-Net based) Encoder_skips = [] Current_features = F_recombined For each encoder_stage: Current_features = ConvReXt_Module(Current_features) Current_features = DGA_Module(Current_features) Add Current_features to Encoder_skips If not last_encoder_stage: Current_features = ConvReXt_Downsampling_Module(Current_features) Bottleneck Bottleneck_features = Current_features 3. Decoder Path Current_features = Bottleneck_features For each decoder_stage: Current_features = Upsample_Module(Current_features) Skip_features_processed = Dense_Multiscale_Module(Encoder_skips.pop()) Current_features = Concatenate(Current_features, Skip_features_processed) Current_features = Dense_Multiscale_Module(Current_features) // Decoder feature refinement 4. Output Segmentation Heads S_wt = Output_Conv_WT(Current_features) S_tc = Output_Conv_TC(Current_features) S_et = Output_Conv_ET(Current_features) Return S_wt, S_tc, S_et End Procedure

The core basis for the design of the multimodal feature recombination module is that the discriminative features of different sub-regions of brain tumors (WT/TC/ET) are highly correlated with the channel response of a specific modality. For example, on the BraTS dataset, the identification of enhanced tumors (ET) strongly depends on the contrast enhancement features of the T1ce modality, while the detection of peritumoral edema (WT-ED) relies more on the high edema signal of the FLAIR modality. Therefore, channel attention needs to adaptively learn the weights of each modality to directly strengthen the modality features that are sensitive to the target region and avoid redundant calculations of spatial attention due to the randomness of tumor location. This targeted fusion of modality features also alleviates the differences in different tumor sub-regions in different modalities and the class imbalance problem that may be exacerbated by this. Although we did not use a dedicated loss function to directly deal with class imbalance, the design of the multi-task learning framework enables each subtask (WT, TC, ET segmentation) to independently optimize the segmentation performance of its corresponding region, which helps the model better learn and identify small target regions. In contrast, although graph-based methods can model the relationship between modalities, their high computational complexity makes it difficult to apply to three-dimensional medical images.

To address the severe class imbalance challenge arising from the minimal or even absent active tumor regions in brain tumor data, particularly in low-grade gliomas (LGG), the proposed multi-task learning framework serves as an architectural mitigation strategy. Rather than configuring five categories within a global Softmax space for competitive classification—an approach that frequently causes the gradients of small target regions to be overwhelmed by the dominant background—this study decouples the segmentation process into three interconnected yet independent binary classification sub-tasks: whole tumor (WT), tumor core (TC), and enhancing tumor (ET). This decoupling mechanism enables the network to compute losses and optimize feature representations independently for minority target regions. In conjunction with the Multimodal Feature Recombination Module (MRM), which adaptively amplifies the feature weights of specific sensitive modalities, the model effectively increases the signal-to-noise ratio of minority classes at the feature level. Consequently, this approach improves the model’s robustness against severely imbalanced data without necessitating the introduction of additional complex class-weighting algorithms.

**Figure 1:**
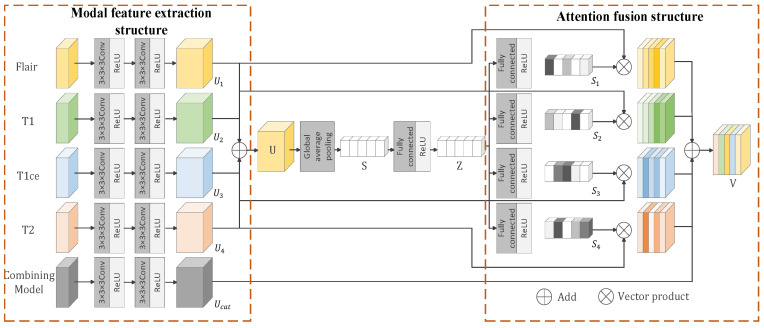
Structural diagram of multimodal feature recombination module.

To fully extract complementary features from multi-modal MRI images, this paper proposes the MR module, as shown in Figure 1, based on the SK-Net network’s [19] idea of adaptively selecting feature branches. This module can be divided into two processing stages. First, the features of the four MRI modality images (Flair, T1, T1ce, T2) are extracted respectively, and the preliminary feature maps are obtained through convolution and activation operations, and then fused into shared feature maps. Subsequently, the module constructs the attention channel weights through global average pooling and fully connected layers, generates weight coefficients using the Softmax function, and then performs weighted combination with the feature maps of each modality to obtain the final fusion output. The left side of the figure shows the modality input path, and the right side reflects the differential fusion mechanism under the guidance of channel attention.

For the four-modal MRI medical images, feature extraction is performed for each modality using 16 3D convolutional kernels of size 3 and the ReLU activation function, resulting in feature maps $\left[U_{1},U_{2},U_{3},U_{4}\right]$ for each modality.

(1) $$\begin{array}{c} \;U=\sum_{i=1}^{4}\;U_{i}\; \end{array}$$

Specifically, the c-th element of S is computed by applying spatial compression to the feature map U with dimensions H×W×D:

(2) $$\begin{array}{c} \;S_{c}=F_{GAP}\left(U_{c}\right)=\frac{1}{H\times W\times D}\sum_{i=1}^{H}\;\sum_{j=1}^{W}\;\sum_{k=1}^{D}\;U_{c}\left(i,j,k\right) \end{array}$$

In the formula, $F_{GAP}$ is the global average pooling layer; $U_{c}$ is the element of the c-th channel of the feature map U; and H, W, and D are the height, width, and depth of the feature map, respectively.

Then, in order to learn the attention weights for each modality, a vector Z was created, and nonlinear feature mapping was achieved through fully connected layers and ReLU activation functions:

(3) $$\begin{array}{c} \;Z=\delta \left[F_{FC}\left(S\right)\right] \end{array}$$

In the equation, F is the fully connected layer, and δ is the ReLU activation function.

Next, guided by the feature description vector Z, the Softmax function is used to adaptively assign soft attention weights to feature information of different modalities in the channel dimension:

(4) $$\begin{array}{c} \;X=F_{FC}\left(Z\right) \end{array}$$

(5) $$\begin{array}{c} \;S_{i,c}=\frac{\exp\left(X_{i,c}\right)}{\sum_{j=1}^{4}\;\exp\left(X_{j,c}\right)} \end{array}$$

In the formula, $S_{i,c}$ is the c-th element in $S_{i}$, and $X_{i,c}$ is the c-th element in $X_{i}$.

Perform vector product operation between each modal feature $U_{i}$ and the calculated attention weight $S_{i}$ to obtain a feature map for adjusting weights for subtasks. Finally, the final reconstructed feature V is obtained by summing the features $U_{cat}$ extracted by the four modal superposition methods:

(6) $$V_{c}=U_{cat,c}+\sum_{i=1}^{4}\;S_{i,c}\cdot U_{i,c}\;$$

where $V_{c}$ and $U_{cat,c}$ denote the feature planes of the final recombined feature and the concatenated feature in the c-th channel, respectively; $S_{i,c}$ represents the attention weight scalar of the *i*-th modality in the c-th channel; and $U_{i,c}$ denotes the input feature map of the *i*-th modality in the *c*-th channel.

From an information-theoretic perspective, the multimodal feature recombination module achieves substantial information gain via the attention mechanism. Unweighted baseline concatenated features typically contain redundant background noise and interference from irrelevant modalities, which theoretically increases the uncertainty of the target region. By computing channel weights and performing non-linear mapping, the multimodal feature recombination module maximizes the mutual information between the weighted recombined features and the target tumor sub-regions. This weight allocation mechanism functions as an adaptive information filter. By suppressing noise transmission from low-correlation modalities, it increases the density of effective feature information transmitted to subsequent network layers, thereby providing a theoretical formulation of information gain to support the accurate segmentation of distinct brain tumor sub-regions.

To more comprehensively evaluate the proposed MRM, we also considered other advanced multimodal fusion strategies, such as graph neural network (GNN)-based methods. Such methods usually treat different modalities as nodes in a graph, learn and update the relationship between nodes through an attention mechanism, and explicitly model the complex dependencies between modalities. Although attention-based GNNs can theoretically capture rich inter-modal correlations, they face two major challenges when applied to 3D medical image segmentation: first, building a graph and passing information between nodes will introduce huge computational overhead, resulting in a sharp increase in training time and hardware resource requirements; second, the performance of GNNs is highly dependent on the definition of the graph structure, and defining the optimal graph structure for multimodal MRI is itself an open problem. In contrast, the proposed MRM implicitly learns the contribution weight of each modality to the final segmentation task in a more lightweight and end-to-end manner through a channel attention mechanism. This design is not only computationally efficient, but also directly optimizes for the prior knowledge that brain tumor subregions (e.g., ET, WT) are highly correlated with specific modalities (e.g., T1ce, FLAIR), and has been proven to be a practical strategy that strikes a good balance between accuracy and efficiency.

### 2.2. ReU Net Network Model

#### 2.2.1. Downsampling Convrext Module

**Figure 2:**
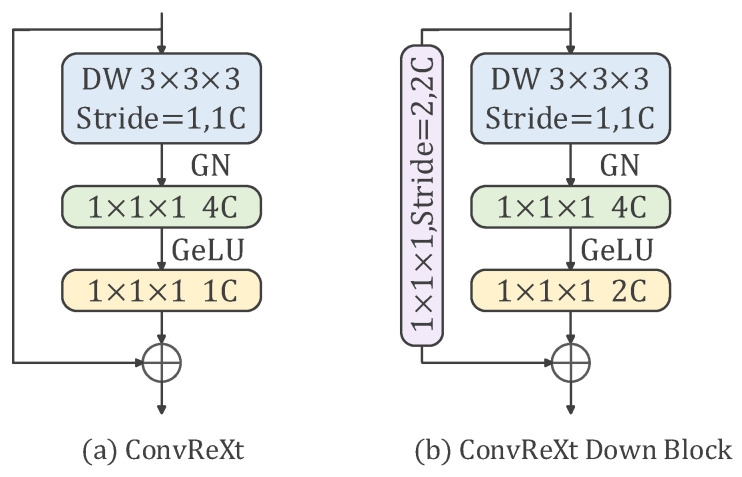
Structure diagram of ConvReXt and ConvReXt Down Block. Unlike the relatively simple 2 × 2 convolutional downsampling layer in the original ConvNeXt, the proposed module designs the downsampling step itself as a more complex unit: it first uses a depthwise separable convolution with a stride of 2 to reduce the spatial size of the feature map and perform preliminary feature extraction. Next, the downsampling module directly integrates the reverse bottleneck design, and the channel width is first expanded to four times the number of input channels to introduce stronger nonlinear expression capabilities and capture richer features [21]. Subsequently, in the compression layer, the channel width is adjusted to twice the number of input channels. This approach of directly integrating depthwise separable convolution and a specific ratio of reverse bottlenecks in the downsampling unit is a specialization and enhancement of the original ConvNeXt downsampling method. In order to effectively implement residual learning during the downsampling process, the module’s shortcut connection is also specially designed through a 1 × 1 convolution with a stride of 2, and the channel width is expanded to twice the number of input channels to match the number of output channels after the main path is downsampled, ensuring smooth transmission and effective fusion of information. Through this series of careful designs, the proposed ConvNeXt downsampling module can more effectively retain and enhance semantic information while reducing the resolution of feature maps, which is crucial for tasks that require precise detail information such as medical image segmentation, and is expected to bring significant performance improvements.

Compared with traditional pooling operations that may cause information loss when reducing the spatial resolution of feature maps [20], convolutional downsampling reduces the size of feature maps by retaining local spatial information, thereby more effectively preserving details, while its trainable convolution kernels enable the model to learn more complex and abstract feature representations. Traditional convolutional downsampling methods sometimes fail to fully utilize the potential of channel width, while increasing channel width can significantly enhance the nonlinear characteristics of the network, thereby improving its expressive power and ability to extract semantic information. Inspired by the ConvNeXt architecture and combined with the reverse bottleneck design idea from Transformer, this paper proposes an improved ConvNeXt downsampling module (as shown in Figure 2) to optimize the feature processing process, which is particularly suitable for tasks that are sensitive to detail information such as medical image segmentation. The original ConvNeXt model borrows many designs from the Vision Transformer in its core module, such as the use of depthwise separable convolutions and reverse bottleneck structures (i.e., the hidden dimension of the MLP block is four times the input dimension). Its standard downsampling operation is implemented using a separate 2 × 2 convolutional layer (with a stride of 2) between stages, usually preceded by LayerNorm for normalization. A key design in Transformer is also the reverse bottleneck structure within the MLP block, that is, the dimension of the intermediate layer is much larger than the input/output dimension.

Traditional convolutional downsampling methods often fail to fully exploit the potential of channel width. Increasing the channel width can significantly enhance the network’s nonlinearity, thereby improving its expressive power and ability to extract semantic information [22]. Based on ConvReXt, this paper introduces an improved ConvReXt downsampling block, incorporating the inverse bottleneck design from Transformers, as shown in Figure 2. In this module, the process of optimizing feature handling is achieved through several key steps. First, depthwise separable convolution layers are used with a convolution stride of 2 to reduce the spatial dimensions of the feature map. Next, the inverse bottleneck structure is employed to expand the channel width to four times the input width. This design introduces more nonlinearity, thereby enhancing the network’s learning capability. Subsequently, in the compression layer, the channel width is adjusted to twice the input width. Finally, to ensure the effectiveness of residual connections, a 1 × 1 × 1 convolution with a stride of 2 is added in the connection section, and the channel width is expanded to twice the input width, ensuring smooth information flow and fusion. Through this series of designs, the ConvReXt downsampling block is able to retain more semantic information in low-resolution feature maps, significantly improving the performance of medical image segmentation tasks.

To further improve the reproducibility of the ConvReXt downsampling module, its key structural parameters are supplemented. This module uses depthwise separable convolution to reduce redundant calculations and enhance feature extraction capabilities. The channel expansion ratio is set to 4 times to introduce more nonlinear transformation space, and then the number of channels is compressed to twice the number of input channels in the compression stage to ensure the compactness and richness of feature expression. The stride of all downsampling convolution operations is uniformly set to 2 to achieve a reduction in spatial resolution while retaining key semantic information. In order to ensure the dimensionality matching of the residual connection, a 1 × 1 × 1 convolution operation with a stride of 2 is introduced in the residual path, and the number of channels is expanded to twice the input, thereby achieving effective fusion of the main branch path information. The above parameter settings have been verified by preliminary experiments, significantly improving the segmentation performance while maintaining network stability.

#### 2.2.2. Dense Parallel Global Spatial Attention Module

**Figure 3:**
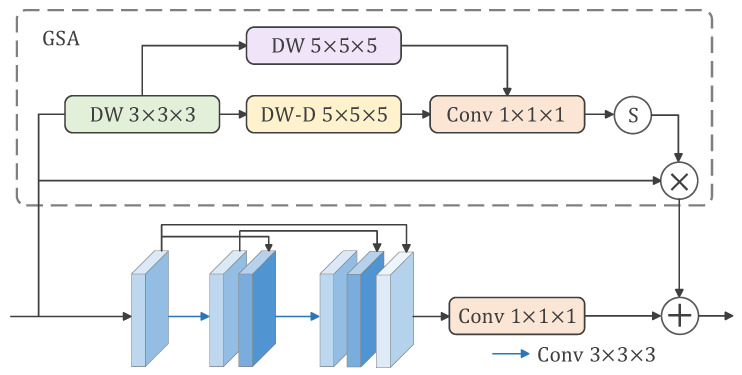
Structure diagram of dense parallel global spatial attention module. The module consists of two parallel branches. The first branch uses the dense block (DSN) from the DenseNet architecture to prevent gradient vanishing and facilitate feature reuse. Considering the large number of parameters in 3D convolutions, the number of layers in the dense block is set to 2 to effectively control the computational load. The input to each layer is the output of all preceding layers, and the features extracted at each layer are passed to subsequent layers. Finally, the number of channels is adjusted through a 1 × 1 × 1 convolutional layer. This feature propagation process is represented by Equation (7).

In image segmentation tasks, it is crucial for the network to possess the ability to model both remote and local features. Traditional convolutional neural networks (CNN) have achieved mature results in local feature modeling, but they exhibit significant limitations in handling long-range dependencies. Currently, there are two main approaches used to capture remote information in images. The first approach is to introduce self-attention mechanisms to capture long-range dependencies. However, its quadratic complexity results in a large number of network parameters and computational overhead, and this method often simplifies images from multi-dimensional forms to one-dimensional sequences, which in turn disrupts the spatial structure of the image. The second approach involves modeling long-range dependencies using large-kernel depthwise convolutions. Compared to self-attention mechanisms, large-kernel convolutions better preserve the spatial structural information of the image and effectively model the dependencies between local and remote information. However, as the size of the convolutional kernel increases, the computational cost also rises significantly. To address this issue, VAN proposes an innovative solution that decomposes the large convolutional kernel into three components: depthwise convolution, dilated convolution, and channel convolution (1 × 1 convolution). Although this method improves computational efficiency to some extent, dilated convolutions suffer from sparsity in feature extraction and lack multi-scale receptive fields, leading to room for optimization in VAN-based segmentation methods. To further enhance performance, this paper designs a Dense Parallel Global Attention (DGA) module, as shown in Figure 3, aimed at addressing the above issues and improving the modeling of long-range information.

(7) $$\begin{array}{c} \;X_{l}=N\left(\left[X_{0},\ldots ,X_{l-1}\right]\right) \end{array}$$

Among them, $X_{l}$ represents the output of the l-th layer, $X_{0}$, …, $X_{l-1}$ represents the concatenation operation, N represents the nonlinear transformation, consisting of 3 × 3 × 3 convolution, normalization, and GELU activation function, and then passing through a 1 × 1 × 1 convolution dimensionality reduction channel to obtain the output $y_{l}$ of a branch.

The introduction of the dense block branch (DSN) theoretically optimizes the gradient propagation dynamics in the deep layers of the network. In deep 3D convolutional networks, conventional sequential architectures are susceptible to the gradient vanishing phenomenon during backpropagation. Leveraging the feature reuse mechanism enabled by dense connections, forward-propagated features can flow directly into subsequent layers, thereby inherently sustaining the gradient flow during backpropagation. According to the chain rule, if the final loss function at a specific stage of the network is denoted as L, the gradient of this loss with respect to the early shallow input features $X_{0}$ can be derived as expressed in Equation (8):

(8) $$\begin{array}{c} \;\frac{\partial L}{\partial X_{0}}=\frac{\partial L}{\partial X_{l}}\left(1+\frac{\partial }{\partial X_{0}}\sum_{i=1}^{l-1}N\left(X_{0},\ldots ,X_{i-1}\right)\right) \end{array}$$

where *N* represents a non-linear transformation operation consisting of convolution, normalization, and an activation function. The constant term 1 within the parentheses holds an explicit structural significance: it ensures that gradient signals from deep layers can bypass complex non-linear transformation layers without attenuation, thereby establishing a direct backpropagation path to the shallow layers of the network. This theoretical property effectively mitigates the risk of gradient decay introduced by complex attention mechanisms, facilitating the efficient convergence of the model with large-scale parameters while achieving precise updates of local detail features in high-dimensional medical images.

The other branch is the global spatial attention (GSA) branch, which draws on the idea of LKA in VAN to capture long-distance dependencies by decomposing large convolution kernels. LKA usually contains deep convolution (DW-Conv), deep dilated convolution (DW-D-Conv) and 1 × 1 convolution. The GSA branch of this module first uses a 3 × 3 × 3 spatial local convolution (DW-Conv) to extract basic features, and then captures extensive long-range dependencies through a spatial long-distance convolution (DW-D-Conv) with a convolution kernel size of 5 × 5 × 5 and a dilation rate of 2. Its key innovation is that in order to solve the feature sparsity problem that may be caused by dilated convolution and avoid incomplete semantic information extraction, GSA integrates a deep convolution layer with a dilation rate of 1 and a size of 5 × 5 × 5 in parallel next to the above-mentioned spatial long-distance dilated convolution. This design aims to obtain denser context information by fusing features from convolution paths with the same large receptive field but different sparsity. Finally, 1 × 1 × 1 convolution (channel convolution) is used to achieve cross-channel information interaction and enhance the expressiveness of features. This process helps to fuse the outputs of deep dilated convolution and deep convolution and improve the overall performance of the network. This process can be expressed by Formula (9):

(9) $$\mathrm{Att}={\mathrm{Conv}}_{1\times 1\times 1}\left({\mathrm{DWC}}_{\mathrm{dilated}}^{\left(5\times 5\times 5\right)}\left({\mathrm{DWC}}^{\left(3\times 3\times 3\right)}\left(I\right)\right)+{\mathrm{DWC}}_{\mathrm{dense}}^{\left(5\times 5\times 5\right)}\left({\mathrm{DWC}}^{\left(3\times 3\times 3\right)}\left(I\right)\right)\right)\;$$

where I denotes the input feature map to the GSA branch; $DW\text{\_}Conv_{3\times 3\times 3}\;$ refers to the depthwise convolution, with 3 × 3 × 3 indicating the corresponding convolutional kernel size; and Att represents the final output of the entire GSA branch following the aforementioned operations, i.e., the spatial attention map.

The dual-parallel architecture of the GSA branch achieves a theoretical balance between expanding the receptive field and preserving spatial feature density. For a 3D convolutional network, the one-dimensional receptive field $RF_{l}$ of the l-th layer is calculated as follows:

(10) $$\begin{array}{c} \;RF_{l}=RF_{l-1}+\left(k-1\right)\times d \end{array}$$

where k denotes the convolutional kernel size and d represents the dilation rate. In a conventional single-pathway dilated convolution, a single 5 × 5 × 5 depthwise dilated convolution with d=2 can efficiently expand the receptive field to a spatial extent of 9 × 9 × 9. However, its strided sampling mechanism results in a grid-like sparse distribution of the effectively activated voxels. This study introduces a parallel standard large-kernel branch with d=1, which possesses a theoretical receptive field of a continuous 5×5×5 region. Through spatial feature summation, the combination of these two branches not only ensures the network’s capability to capture long-range anatomical dependencies but also theoretically fills the inactive 3D spatial blind spots generated by the dilated convolution, thereby achieving comprehensive and dense coverage of the contextual information within the lesion region.

To ensure the interpretability and reproducibility of the DGA module structure design, this paper explains the settings of the main hyperparameters as follows: the dense block part is set to 2 layers, aiming to improve the efficiency of feature reuse and prevent gradient disappearance under the premise of controlling the number of parameters; the number of output channels of each layer is set to be consistent with the input to maintain the stability of feature dimensions. 1 × 1 × 1 convolution is used in channel convolution for dimension compression and cross-channel information fusion, and its structure refers to the typical design in DenseNet and lightweight attention mechanism. In the global spatial attention branch, the 5 × 5 × 5 convolution layer with a dilation rate of 2 is used to expand the receptive field, while the parallel convolution of the same size with a dilation rate of 1 is used to alleviate the problem of feature sparsity. The above parameters have been pre-experimentally tuned to ensure the rationality of the model structure design and the improvement of the actual segmentation effect without significantly increasing the complexity of the model.

On this basis, the attention weights of global information are obtained through the Sigmoid activation function. The attention weights are multiplied by the input branch I at the element level and fused globally with the original feature map to obtain the feature map $y_{2}$. This effectively achieves local information and remote connections, promoting spatial fusion.

(11) $$\begin{array}{c} \;y_{2}=I⊗\sigma \left(Att\right) \end{array}$$

Among them, σ represents the Sigmoid activation function. Based on the above two branches, the final output of the module is obtained by adding the output $y_{1}$ of the first branch and the output $y_{2}$ of the second branch at the element level:

(12) $$\begin{array}{c} \;Y_{DGA}=y_{1}+y_{2} \end{array}$$

In summary, the proposed Multimodal Feature Recombination Module (MRM) and the Dense Parallel Global Attention (DGA) module establish an orthogonal and highly synergistic working mechanism architecturally. They theoretically optimize the network from two core dimensions of feature representation: the MRM operates in the channel dimension to address the issue of “what to attend,” adaptively refining the effective signals of various MRI physical modalities through cross-modal interactions; the DGA operates in the spatial dimension to address the issue of “where to attend,” accurately localizing lesion boundaries and capturing global context through the parallel synergy of large-kernel dilated convolutions and dense blocks. This cascaded synergistic paradigm—combining channel filtering with dense spatial focusing—ensures that the input features received by the DGA module are modality-optimized and highly discriminative representations. Consequently, it maximizes the efficacy of the spatial attention mechanism, ultimately achieving a comprehensive enhancement in the performance of multi-task brain tumor segmentation.

#### 2.2.3. Dense Multi-Scale Module

**Figure 4:**
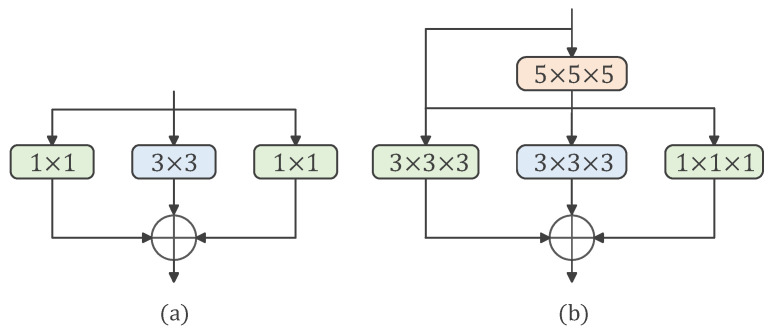
(**a**) Inception (**b**) Dense Multi-scale Module (DDM). The core innovation of DDM (Dense Multi-scale Module) lies in its unique structural design and its application in the network. It aims to enhance the semantic transformation ability of the skip connection path and the decoder unit itself, so as to more effectively fuse feature information at different levels. Specifically, DDM draws on the multi-branch parallel structure of the Inception module and uses convolution kernels of different sizes (5 × 5, 3 × 3 and 1 × 1 in this paper) to process input features simultaneously, which enables the module to capture and fuse contextual information of multiple scales at the same level. At the same time, the entire DDM follows the residual learning concept of ResNet in design. Its output is a combination of the multi-branch convolution processing results and the input features passed through the shortcut connection, which helps to optimize gradient propagation and promote model training. Compared with the original skip connection of U-Net or the simple use of standard ResNet modules in the skip connection, DDM provides richer feature perspectives and stronger multi-scale information fusion capabilities through its parallel multi-scale convolution kernel design. Innovatively, DDM is directly applied to the skip connection path of the network. Before the encoder features enter the decoder, DDM processing can convert the original encoder features that are rich in details but low in semantic level into representations containing richer context and multi-scale information, thereby more accurately matching the needs of the corresponding level of the decoder at the semantic level. In addition, DDM is also applied to various stages of the decoder. In the decoder, DDM, with its multi-scale characteristics, helps the network to gradually restore the spatial resolution while more effectively integrating the features transmitted by the previous decoding layer and the skip connection enhanced by DDM, achieving more accurate boundary positioning and region recognition.

In the traditional encoder–decoder architecture, the encoder is responsible for converting input data into feature representations, but these features are usually low-level features due to the shallow convolutional layers; while the decoder is responsible for restoring these features to the final output. The features extracted in the decoder are usually high-level because its convolutional layers are deep, so there is a significant semantic difference between the encoder and the decoder. In addition, an obvious limitation of the encoder–decoder structure is that the context information in the encoding stage is the same at each time step, which may become a bottleneck for model performance. To overcome this problem, this paper aims to make the decoding process rely on more relevant context information, so an encoder and decoder different from the traditional structure are designed, which can further expand the semantic difference between the encoder and the decoder. In existing methods, some networks, such as UNetR and Swin-UNETR, use ResNet structures to improve skip connections and decoders, thereby reducing the semantic gap between the encoder and the decoder and improving decoding capabilities. However, the use of simple ResNet modules in skip connections still has certain limitations, especially in terms of semantic conversion capabilities. Therefore, there is room for further optimization of the skip connection and decoder structure based on ResNet. To this end, this paper combines the Inception and ResNet structures and proposes a new dense multi-scale module, as shown in Figure 4.

#### 2.2.4. Loss Function

Binary Cross Entropy (BCE) is commonly used as the loss function in medical image segmentation, as shown in Formula (13):

(13) $$\begin{array}{c} \;L_{bce}\left(G,P\right)=-\frac{1}{I}\sum_{i=1}^{I}\;\sum_{j=1}^{J}\;G_{i,j}\log P_{i,j} \end{array}$$

where I represents the number of voxels; J represents the number of categories; and $P_{i,j}$ and $G_{i,j}$ respectively represent the probability output and true value of the j-th class on the *i*-th voxel.

A class imbalance problem exists in brain tumor segmentation tasks, where the background region is substantially larger than the overall tumor region, and the peritumoral edema region is significantly larger than the necrotic and enhancing tumor regions. The standard Binary Cross-Entropy (BCE) loss computes the loss on a pixel-by-pixel basis, assigning equal weights to both foreground and background. Consequently, the model is prone to being dominated by background pixels during training, yielding background-biased predictions and overlooking small lesions such as enhancing tumors. To mitigate this data imbalance issue, this study introduces the Dice loss function in conjunction with the BCE loss, as formulated in Equation (14):

(14) $$\begin{array}{c} \;L_{Dice}\left(G,P\right)=1-\frac{2}{J}\sum_{j=1}^{J}\;\frac{\sum_{i=1}^{I}\;G_{i,j}P_{i,j}}{\sum_{i=1}^{I}\;G_{i,j}^{2}+\sum_{i=1}^{I}\;P_{i,j}^{2}} \end{array}$$

Therefore, the total loss is:

(15) $$\begin{array}{c} \;L\left(G,P\right)=L_{bce}\left(G,P\right)+L_{Dice}\left(G,P\right) \end{array}$$

Brain tumor segmentation tasks inherently suffer from severe class imbalance, where regions with minimal volumes, such as the enhancing tumor, are highly susceptible to being overwhelmed by the dominant background class, leading to under-segmentation. To address this primary challenge, this study proposes a systematic, multi-dimensional synergistic strategy to mitigate class imbalance from both data and architectural perspectives. This paradigm serves as a robust alternative to Focal Loss or static class weighting strategies, which are hyperparameter-sensitive and vulnerable to noise from ambiguous medical image boundaries. In terms of data augmentation, the random spatial cropping employed during the training phase significantly increases the sampling frequency of lesion regions within the input patches. Architecturally, the model decouples the complex segmentation into three independent binary classification sub-tasks, thereby isolating the gradients of minute lesions from the suppressive influence of the background class. Concurrently, the Multimodal Feature Recombination Module (MRM) adaptively amplifies the signals of sensitive modalities, effectively enhancing the signal-to-noise ratio of small lesions at the shallow layers of the network.

Supported by this systematic framework, the total loss function jointly incorporates Binary Cross-Entropy (BCE) and Dice losses. The Dice loss optimizes the spatial overlap between the predicted segmentations and the ground truth, and its formulation is inherently insensitive to the absolute quantity of background pixels. By integrating these two objectives, the total loss preserves the gradient stability of BCE in pixel-wise classification while leveraging Dice loss to mitigate class imbalance, thereby sustaining the network’s capability to identify small-volume targets such as active tumor regions. This synergistic strategy effectively minimizes the risk of omitting minute lesions without introducing additional hyperparameters.

## 3. Experimental Results and Analysis

### 3.1. Dataset and Experimental Settings

**Figure 5:**
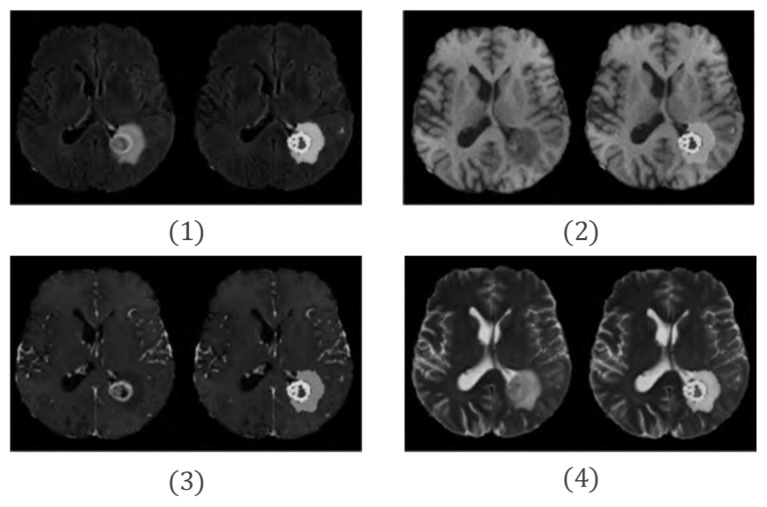
FLAIR, T1, T1ce, T2 sectional view. From left to right, and top to bottom, the axial images of FLAIR, T1, T1ce, and T2 modalities of a particular case are displayed. (**1**) FLAIR: This modality suppresses the high signal from cerebrospinal fluid and highlights areas of brain edema. Regions with higher water content appear brighter in the image, facilitating the identification of edema surrounding the tumor. (**2**) T1: This modality is primarily used to observe brain anatomy. Although it helps in identifying anatomical landmarks, it is less effective in clearly revealing lesions. (**3**) T1ce: A contrast agent is injected into the patient before MRI, enhancing the contrast in areas of active blood flow and making the enhancing tumor regions more distinct. This modality is commonly used for tumor identification. (**4**) T2: This modality provides a clearer depiction of tumor lesions, making it particularly suitable for overall tumor assessment. On the right side of each image group is the corresponding label image. The darkest central area represents the tumor core (TC), the immediate surrounding area is the outer ring of the tumor core (TC), and the largest outer area indicates the peritumoral edema region (ED).

The dataset utilized in this study is obtained from the Brain Tumor Segmentation (BraTS) 2020 public benchmark. This dataset comprises preoperative MRI scans from 335 glioma patients, consisting of 76 low-grade glioma (LGG) and 259 high-grade glioma (HGG) cases. The dataset is partitioned into training and testing cohorts with a ratio of 80:20. Each patient case includes four MRI modalities: T1-weighted (T1), T1-weighted contrast-enhanced (T1ce), T2-weighted (T2), and Fluid-Attenuated Inversion Recovery (FLAIR). Additionally, manual ground truth annotations labeled by experts are provided for each case. The spatial dimension of each MRI volume is 240 × 240 × 155 voxels. The tumorous areas are categorized into three distinct sub-regions: peritumoral edema (ED), tumor core (TC), and enhancing tumor (ET), which are combined to constitute the whole tumor (WT) area. A schematic representation of the multi-modal MRI data and the corresponding annotations is illustrated in Figure 5.

During the data partitioning stage, to eliminate the risk of data leakage, a five-fold cross-validation scheme is implemented strictly at the patient level. This strategy ensures that all multi-modal volumes and their corresponding randomly cropped patches from a single patient are strictly assigned to either the training fold or the validation/testing fold, thereby avoiding cross-contamination. In the data preprocessing phase, all volumetric data are randomly cropped to a target patch size of 128 × 128 × 128 voxels. Intensity normalization is performed using the Min-Max standardization method. To further prevent statistical feature leakage, the computation of the minimum ($I_{MIN}$) and maximum ($I_{MAX}$) intensity parameters for each modality is strictly confined within the training cohort of the current fold. Specifically, for each MRI modality (T1, T1ce, T2, and FLAIR), $I_{MIN}$ and $I_{MAX}$ are first calculated across the training set. Subsequently, the intensity value I of each voxel is uniformly scaled according to Equation (16). These fixed extreme parameters are then applied to normalize the corresponding validation and testing cohorts. This strict isolation mechanism guarantees that the testing data remains completely unseen during the model training phase. The transformation is executed as expressed in Equation (16):

(16) $$\begin{array}{c} \;Dice\left(G,P\right)=\frac{2\sum_{i=1}^{I}\;G_{i}P_{i}}{\sum_{i=1}^{I}\;G_{i}+\sum_{i=1}^{I}\;P_{i}} \end{array}$$

This normalization method helps to eliminate the intensity differences between different modalities and ensures that the network input data has a consistent numerical range, thereby improving the training stability and convergence speed of the model.

### 3.2. Experimental Environment and Parameter Design

The proposed network model is implemented using Python 3.7 and PyTorch 1.11.0, and the experiments are conducted on an NVIDIA RTX 3090 GPU. A five-fold cross-validation with strict patient-level isolation is applied to evaluate the 335 samples. In each fold, the independent test set contains 67 samples. The remaining 80% of the dataset is further partitioned into an internal training set (241 cases) and an internal validation set (27 cases) at a 9:1 ratio to structurally prevent data leakage. During the training phase, input images are randomly cropped to 128 × 128 × 128 voxels to augment sample diversity. The Adam optimizer is utilized with a batch size of 1 and a weight decay of 1 × 10^−5^. A dropout layer with a rate of 0.2 is incorporated into the network. The initial learning rate is set at 1 × 10^−3^ and modulated by a cosine annealing decay strategy. The maximum number of training epochs is capped at 400. To synergistically mitigate model overfitting, an early stopping mechanism is triggered by monitoring the loss variation in the internal validation set.

This study utilizes a five-fold cross-validation strategy to evaluate the 335 BraTS 2020 samples. In each experimental fold, the dataset is divided into a training subset and an independent testing subset at a 4:1 ratio. To facilitate the early stopping method and prevent test data leakage, the training subset is further split into an internal training set and an internal validation set at a 9:1 ratio. Specifically, the internal training set (241 samples) is used to update the network weights; the internal validation set (27 samples) is utilized to monitor the loss function during training and trigger the early stopping mechanism. The remaining 67 samples serve as the independent test set, exclusively reserved for evaluating the segmentation performance of the final model. This partitioning scheme ensures the objectivity of the model selection and evaluation processes. The training process is accelerated using a GPU.

### 3.3. Model Evaluation Indicators

In this study, the segmentation algorithm is used to delineate three tumor regions in the patient’s multimodal MRI images: WT, TC, and ET, where WT includes TC, and TC includes ET, forming a hierarchical nested topological structure. To evaluate the performance of the model in the segmentation task, this study employs the Dice coefficient, sensitivity, and the 95% Hausdorff distance as evaluation metrics.

The Dice coefficient is the primary metric for assessing segmentation accuracy, reflecting the overlap between the model’s predicted region and the ground truth annotations. Its value ranges from 0 to 1, where 0 indicates no overlap and 1 indicates complete overlap. In tumor segmentation tasks, the Dice coefficient provides a clear visualization of the match between the predicted region and the actual region, serving as a crucial metric for evaluating segmentation accuracy.

Sensitivity is used to measure the model’s ability to correctly identify the true tumor region, specifically the proportion of pixels in the actual tumor region that are correctly labeled. In clinical practice, failing to detect the true tumor region may result in incomplete treatment. Therefore, sensitivity is a key metric for evaluating the model’s ability to prevent false negatives.

The 95% Hausdorff Distance (HD) is used to assess the model’s accuracy in segmenting tumor boundaries. This metric calculates the maximum distance between the predicted and true boundaries, rigorously testing the model’s ability to capture tumor edge details. The 95% Hausdorff Distance considers the 95th percentile of the distances between all point pairs, reducing the impact of outliers and providing a more accurate reflection of the model’s performance in most cases.

(17) $$\begin{array}{c} \;Sensitivity\left(P,T\right)=\frac{|P\wedge T|}{|T|} \end{array}$$

(18) $$HD(G^{\prime },P^{\prime })\&=\max\left\{\begin{array}{c} \underset{g^{\prime }\in G^{\prime }}{max}\;\underset{p^{\prime }\in P^{\prime }}{min}\;∥g^{\prime }-p^{\prime }∥,\\ \underset{p^{\prime }\in P^{\prime }}{max}\;\underset{g^{\prime }\in G^{\prime }}{min}\;∥p^{\prime }-g^{\prime }∥ \end{array}\right\}\;$$

Among them, G represents the lesion area predicted by the model, P represents the actual lesion area, ∧ represents the logical “AND” operation, $G_{i}$ and $P_{i}$ respectively represent the true value and probability output on the i-th voxel, and $G^{\prime }$ and $P^{\prime }$ represent the true value and predicted surface point set.

### 3.4. Ablation Experiment

#### 3.4.1. Multimodal Feature Recombination Module

In order to verify the effectiveness of each design module in the proposed method, we conducted ablation experiments on the basic model 3D-UNet, and investigated the effects of introducing the multimodal feature reorganization module and the dense parallel global attention module. The experimental results are shown in Table 1. From the data in Table 1, it can be seen that after adding the multimodal feature reorganization module to the 3D-UNet model, by assigning appropriate weights to each modality, the model can more effectively fuse information from different modalities, thereby improving the segmentation performance of each tumor lesion area, especially the Dice coefficient of the enhanced tumor (ET) area is improved by 7.33%.

As observed from the evaluation metrics in Table 1, the Hausdorff distance for the whole tumor (WT) region is generally higher than that for the enhancing tumor (ET) region. This phenomenon is primarily attributed to the pathological and imaging characteristics of brain tumors. The WT encompasses the peritumoral edema region, which exhibits diffuse infiltration into normal brain tissue, resulting in highly ambiguous and irregular boundaries. Since the Hausdorff distance is highly sensitive to the maximum error along the predicted boundaries, outlier predictions near the ambiguous boundaries of the edema region are prone to inducing substantial distance errors. Conversely, the ET region presents high contrast in the T1ce modality, yielding relatively distinct and sharp boundaries. Consequently, its boundary localization is more precise, leading to a relatively lower Hausdorff distance. The incorporation of the Multimodal Feature Recombination Module (MRM) effectively reduces the Hausdorff distance across all sub-regions, further demonstrating the advantages of adaptive feature fusion in improving the localization of ambiguous boundaries.

Through its adaptive channel weighting mechanism, MRM enables the network to learn the most discriminative modal features for the current segmentation task, providing a purer and more focused input for subsequent feature extraction (including ConvReXt and DGA). This modality-level optimization can effectively avoid the interference of irrelevant modal information on downstream feature learning, thereby laying the foundation for the DGA module to more accurately capture local and long-range dependencies. Furthermore, after adding the dense parallel global attention module, all evaluation indicators of the model are significantly improved through the fusion of multi-scale features and a wider receptive field. When these two modules are applied simultaneously, the model can fully combine the advantages of both and further improve the overall performance. The experimental results show that the proposed multimodal feature recombination module and dense parallel global attention module have obvious effects in the brain tumor segmentation task.

#### 3.4.2. ConvReXt Module

The ConvReXt module consists of a 3 × 3 × 3 depthwise convolution and a 1 × 1 × 1 depthwise convolution. According to the experimental results in Table 2, removing the 3 × 3 × 3 and 1 × 1 × 1 convolution layers from ConvReXt led to a significant decrease in the Dice coefficient. In particular, after removing the 3 × 3 × 3 depthwise convolution, the Dice coefficients for the WT, TC, and ET regions decreased by 1.25%, 2.11%, and 4.17%, respectively. Further removal of the entire ConvReXt module resulted in a decrease in the Dice coefficients for the three regions by 1.90%, 2.93%, and 8.83%, respectively. These results indicate that the introduction of the ConvReXt module significantly enhanced the expression of local features in the image, providing richer local information for the subsequent Dense Parallel Global Attention Module, which helps improve overall segmentation performance.

#### 3.4.3. Dense Parallel Global Attention Module

From the experimental results in Table 3, it can be seen that when only the global spatial attention (GSA) module is retained, the theoretical receptive field is maintained at 13 × 13 × 13. At this time, for each region of the tumor, the Dice coefficients of WT, TC, and ET reach 89.65%, 86.55%, and 83.46%, respectively. However, when the dense block is directly fused with the output of the GSA module, the Dice coefficients of the three regions are significantly improved, and finally reach the best performance of 93.45%. This shows that DSN effectively compensates for the lack of local detail enhancement of GSA beyond the pure global information capture by promoting feature reuse and enhancing feature propagation. The dense connection characteristics of DSN enable DGA to make full use of the multimodal fusion features after MRM pre-weighted channels. Specifically, MRM assigns different weights to different modal channels in the early stage, highlighting the modal information that is important for lesion identification. When these weighted features enter the DSN branch of DGA, dense connections can ensure that this filtered and enhanced modal information is more effectively transmitted and reused in the deep layer of the network, thereby avoiding the attenuation or loss of important features, and further improving the ability of DGA to capture complex lesion features. In order to further analyze the impact of the internal structure of the GSA module on performance, we removed the 5 × 5 × 5 convolution layer in the GSA module. Experiments show that this modification causes the Dice coefficient of WT, TC, and ET to decrease by 0.30%, 2.46%, and 1.09%, respectively. This result shows that the 5 × 5 × 5 convolution layer plays a key role in the GSA module. It effectively compensates for the sparsity problem of feature extraction that may be caused by dilated convolution, thereby improving the performance of the overall model.

### 3.5. Computational Cost Analysis Experiment

In order to evaluate the practicality of the proposed model in the actual clinical environment, we quantitatively analyzed the computational cost of the proposed model and its key modules, focusing on the number of floating-point operations (FLOPs) and parameters of the model. These indicators are important criteria for measuring model complexity and inference efficiency. We used the thop library to measure the FLOPs and parameters of each module and the overall model under the condition that the input image size is 128 × 128 × 128 voxels. The specific results are shown in Table 4.

As can be seen from Table 1, the computational cost of the proposed model is higher than that of the baseline 3D-UNet, among which the DGA module contributes relatively more to FLOPs and parameters, which is mainly due to its parallel branch design and refined processing of multi-scale features. For example, the computational overhead introduced by the MRM is relatively small, but its contribution to modal feature reorganization is significant, reflecting a high computational benefit. The introduction of the ConvReXt downsampling module also increases some of the computational workload, but it avoids the loss of details caused by traditional pooling by retaining more semantic information.

Although the computational cost of the proposed model exceeds that of the baseline 3D-UNet, in the clinical applicability evaluation, the total time required for our model to complete full-region inference and final reconstruction via a sliding window mechanism for a single comprehensive patient volume (comprising four modalities with 240 × 240 × 155 voxels) is 9.64 s. This processing speed is tailored for non-real-time clinical computer-aided diagnosis scenarios, such as preoperative planning and treatment strategy formulation. In these contexts, clinicians have sufficient time following the examination to analyze the imaging data, negating the necessity for millisecond-level real-time responses. While this inference time precludes application in real-time surgical navigation, it fully satisfies the evaluation criteria of standard clinical workflows. In the future, with advancements in hardware technologies and model lightweighting techniques, the inference speed of the model is anticipated to improve further. This study prioritizes optimizing diagnostic accuracy through enhanced segmentation precision; therefore, the current computational cost represents a justifiable performance trade-off.

Although the number of parameters in the proposed model increases from 16.58 M to 23.86 M—exceeding that of the baseline 3D-UNet—this parameter overhead yields tangible clinical benefits. Experimental results demonstrate that, compared with the state-of-the-art nnFormer model, the proposed model achieves approximately a 1% improvement in segmentation accuracy across all sub-regions. In clinical radiotherapy target volume delineation, a 1% increase in accuracy and sensitivity effectively mitigates the risk of under-segmenting critical regions, such as the enhancing tumor, thereby reducing the probability of tumor recurrence caused by insufficient radiation dosing. Given that the model is primarily intended for non-real-time clinical computer-aided diagnosis tasks—such as preoperative planning and treatment strategy formulation—an inference time of 9.64 s aligns well with standard clinical workflow requirements. Consequently, the current increase in parameter count represents a justifiable technical trade-off, prioritizing high diagnostic accuracy over computational efficiency.

### 3.6. Comparative Experiment

To demonstrate the effectiveness of the proposed algorithm, we compared it with six state-of-the-art CNN methods, including 3D-UNet, Attention UNet [23], UNet++ [24], ET-Net [25], Point-UNet [26], nnU-Net and TransBTS [27]. All of these methods used the same data preprocessing steps and were optimized through hyperparameter tuning to achieve the best results. The experimental results are shown in Table 5.

As shown in the experimental results in Table 5, the proposed model achieved average Dice scores of 92.54%, 89.21%, and 86.54%, which represent improvements of 6.19%, 6.67%, and 5.39%, respectively, compared to the baseline model 3D-UNet. More importantly, compared with advanced Transformer models including nnFormer, our model also shows excellent competitiveness. For example, the Dice scores of our model in the WT, TC, and ET regions are 0.94%, 0.91%, and 1.34% higher than those of nnFormer, respectively. Furthermore, when benchmarked against the highly robust self-configuring framework, nnU-Net, our proposed model maintains a clear structural superiority. Specifically, it outperforms nnU-Net by 0.49%, 0.57%, and 0.92% in the WT, TC, and ET Dice scores, respectively, alongside notable reductions in the Hausdorff distance across all sub-regions. This improvement indicates that the introduction of the multimodal feature recombination module enables the model to more effectively integrate features from different modalities when processing different tumor regions, thereby significantly enhancing segmentation performance for the TC and ET regions, particularly in the more challenging ET region. Additionally, by introducing the scale-cross attention module, the model can fuse information from different scales, capturing more comprehensive and robust feature representations, which further enhances segmentation accuracy. Regarding computational efficiency, while the inference time (9.64 s) is higher than that of the baseline 3D-UNet, it remains notably faster than the resource-intensive nnU-Net (12.85 s). This computational overhead is entirely acceptable considering that the goal of this study is to target non-real-time diagnosis and treatment planning environments. In these application scenarios, segmentation accuracy and reliability are far more critical than real-time performance. It is worth noting that with future advancements in model lightweighting and hardware optimization, the inference speed is expected to improve significantly.

### 3.7. Verification of Generalizability Across Datasets

To evaluate the generalization ability of the model on heterogeneous data, we conducted transfer learning experiments on the BraTS2021 and TCGA-GBM (262 glioblastoma) datasets, as shown in Table 6.

### 3.8. Analysis of Training Dynamics and Convergence

**Figure 6:**
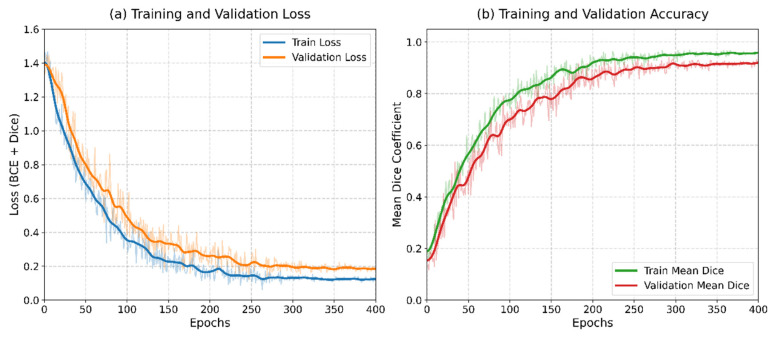
Training and validation curves of the model. (**a**) Loss curves; (**b**) Mean Dice coefficient curves.

Following the validation of the modular effectiveness and the cross-dataset generalization capability, this section further investigates the convergence process and the capacity to mitigate overfitting of the ReU-Net model from the perspective of underlying training dynamics. Figure 6 illustrates the comprehensive training dynamics of the proposed model over 400 epochs during a representative fold of the five-fold cross-validation.

As illustrated by the loss curves in Figure 6a, facilitated by the optimization algorithm and the cosine annealing learning rate strategy during the early stages of training, both the training loss and the validation loss exhibit a rapid and consistent downward trend. After approximately 250 epochs, the two curves gradually converge to a stable plateau. Crucially, the validation loss remains stable throughout the middle and late stages of training, without exhibiting the upward rebound phenomenon typically associated with the continued decrease in training loss. This trajectory visually demonstrates that incorporating an internal validation set coupled with an early stopping mechanism effectively mitigates the risk of overfitting inherent in deep network architectures.

The Mean Dice curves in Figure 6b further corroborate the robustness of the training process. The Mean Dice coefficients for both the training and validation sets increase steadily and achieve convergence at a high plateau, with the validation set ultimately stabilizing within the accuracy range reported in Table 5. A reasonable and stable generalization gap is maintained between the two curves, neither falling into underfitting nor exhibiting an obvious tendency toward the memorization of training features. Given these dynamic characteristics, the proposed architectures—namely, the Multimodal Feature Recombination and the Dense Parallel Global Attention—combined with the joint BCE and Dice loss function, not only excel in the final segmentation metrics but also demonstrate superior training stability throughout the entire parameter optimization process.

## 4. Discussion

**Figure 7:**
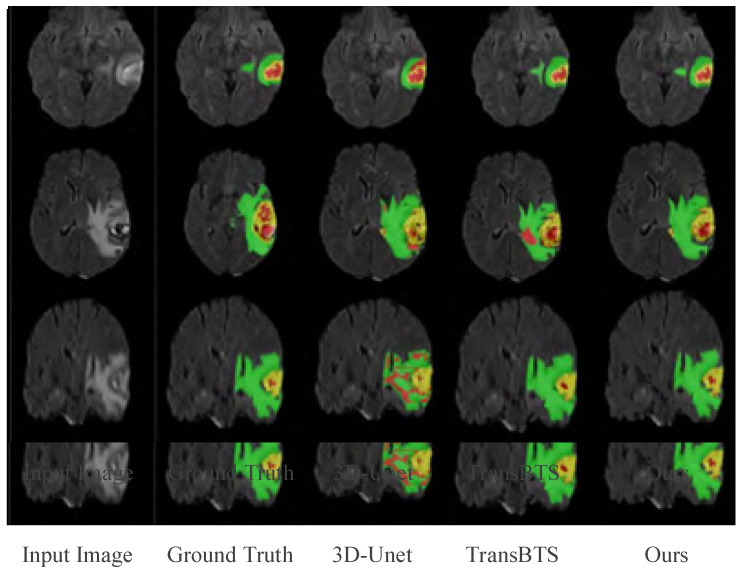
Visualization results of two-dimensional segmentation using different algorithms. It is clearly shown in the figure that the proposed model demonstrates higher segmentation accuracy and completeness when handling irregularly shaped and overlapping brain tumors. Specifically, in the segmentation results of the first brain tumor dataset, the proposed method significantly reduces the occurrence of over-segmentation, with the segmentation results more accurately reflecting the true shape and boundaries of the tumor. In contrast, traditional methods exhibit more over-segmentation or morphological distortion during processing. In the example from the second dataset, the 3D-UNet model shows significant errors in capturing the tumor boundary, whereas the proposed algorithm in this study is able to more accurately identify the tumor contours and their details. Through a comparative analysis of the overall and local segmentation results, it is evident that the proposed method exhibits the highest degree of agreement with the ground truth labels, further validating its superiority and effectiveness in brain tumor segmentation tasks.

To visually demonstrate the advantages of the model in segmentation performance, Figure 7 presents a comparison of segmentation results with other methods. The results indicate that the proposed network achieves higher precision and stability in tumor region recognition, boundary delineation, and detail handling, further validating its superiority and practicality in medical image segmentation tasks.

In the auxiliary diagnosis process, accurate brain tumor segmentation is crucial for subsequent surgical planning and treatment. For example, accurate segmentation of the enhancement tumor (ET) region has a decisive influence on the target area delineation in the radiotherapy plan. If there is a 10% false-negative rate in the ET region, it may lead to insufficient radiation dose, affect the treatment effect and even cause tumor recurrence. Similarly, excessive false positives may lead to over-irradiation of healthy brain tissue, causing unnecessary side effects. The Dice coefficient of the model in this paper reaches 86.54% in the ET region and the sensitivity reaches 86.74%, indicating that it has a high recognition ability for the real tumor region and reduces the risk of false negative rate to a certain extent, thereby supporting more reliable treatment decisions. Although the model’s inference time is 9.64 s, it is not suitable for scenarios with strict speed requirements such as real-time surgical navigation, but for auxiliary clinical diagnosis scenarios such as preoperative planning, radiotherapy planning and disease follow-up, the higher segmentation accuracy it provides can significantly improve the quality of medical care, reduce human errors, and provide a stronger basis for doctors to make accurate decisions.

## 5. Conclusions

Accurate brain tumor segmentation is critical for clinical treatment planning. In this paper, we propose ReU-Net, a 3D deep learning framework based on 3D-UNet for automatic glioma segmentation. A multimodal feature recombination module is designed to adaptively weight multi-modal MRI features and enhance region-specific representation. The ConvReXt module is introduced to reduce information loss during downsampling, and a dense parallel global attention module is employed to capture long-range dependencies and global context.

Ablation experiments verify the effectiveness of each component. Results on BraTS2020 show Dice coefficients of 92.54%, 89.21%, and 86.54% for WT, TC, and ET. In low-grade glioma (LGG) cases, the enhancing tumor (ET) region is typically minimal or absent. The proposed model was cross-validated on a dataset subset comprising 76 LGG cases. To address the absence of this active region, the multi-task decoupled architecture enables the ET branch to output independently; consequently, the absence of this region does not interfere with the feature extraction for the whole tumor (WT) and tumor core (TC). By perceiving the input signals, the Multimodal Feature Recombination Module (MRM) adaptively down-weights contrast-enhanced modalities, such as T1ce, in LGG cases. This mechanism suppresses false-positive predictions in non-active regions, thereby enhancing the network’s adaptability to the pathological characteristics of gliomas across different grades. Despite a mild increase in computational cost, the accuracy improvement is acceptable and practical for non-real-time computer-aided diagnosis.

## Figures and Tables

**Table 1:** Experiments on ablation of different modules. Bold values indicate the optimal segmentation performance for each corresponding evaluation metric.

Models	Mean Dice (%)			Mean Sensitivity (%)			Mean Hausdorff (mm)		
	WT	TC	ET	WT	TC	ET	WT	TC	ET
U-Net	88.72	86.35	79.45	91.68	85.57	84.65	13.45	8.10	5.54
U-Net + MR	89.12	86.45	81.54	91.12	86.12	86.45	10.45	7.87	5.54
U-Net + SC	89.57	**88.12**	83.23	92.45	**88.45**	87.12	8.76	**5.54**	4.26
Ours	**91.12**	86.74	**86.78**	**94.35**	87.65	**91.12**	**5.45**	7.11	**2.45**

**Table 2:** ConvReXt module ablation experiment. Bold numbers represent the optimal experimental results of each column.

Methods	Dice (%)		
	WT	TC	ET
W/O 3 × 3 × 3	90.56	89.54	82.12
W/O 1 × 1 × 1	91.45	91.46	84.66
W/O ConvReXt Block	91.21	90.36	86.78
Ours	**92.46**	**92.47**	**90.95**

**Table 3:** Ablation experiment of dense parallel global spatial attention module. Bolded data denotes the optimal performance per metric.

Methods	Dice(%)		
	WT	TC	ET
W/O DGA	86.87	83.32	83.32
GSA	89.65	86.55	83.46
DSN	90.12	84.13	86.45
W/O 5 × 5 × 5 GSA	89.35	89.01	84.54
Ours	**93.45**	**91.47**	**89.95**

**Table 4:** Computational cost of the model and its key modules.

Model/Module	FLOPs (G)	Parameters (M)
3D-UNet (Baseline)	89.26	16.58
+MRM	91.53	17.21
+ConvReXt Down	98.71	19.34
+DGA	105.89	21.57
Ours	112.45	23.86

**Table 5:** Experimental comparison results of different network models. Bold numbers represent the best results per column.

Models	Mean Dice (%)			Mean Sensitivity (%)			Mean Hausdorff (mm)			Inference Time (s)
	WT	TC	ET	WT	TC	ET	WT	TC	ET	
3D-UNet	86.35	82.54	81.15	90.45	88.37	80.37	11.77	12.87	7.15	4.07
Attention UNet	88.42	85.57	82.47	93.71	87.51	78.53	9.25	12.57	10.04	6.75
UNet++	90.12	86.67	79.57	94.87	93.37	79.04	9.78	9.56	9.68	5.62
ET-Net	89.14	84.58	85.42	94.78	89.57	83.45	6.46	8.71	8.13	4.76
Point-UNet	90.43	88.74	**87.21**	93.45	**94.61**	81.56	5.31	9.55	5.78	7.12
TransBTS	91.32	87.24	84.57	95.72	91.81	85.31	4.34	7.74	6.35	6.54
nnFormer	91.30	88.14	85.87	95.94	92.62	85.46	4.45	6.98	5.93	8.21
nnU-Net	92.05	88.64	85.62	96.28	92.7	85.91	3.98	5.65	5.12	12.85
Ours	**92.54**	**89.21**	86.54	**96.87**	92.73	**86.74**	**3.57**	**5.15**	**4.57**	**9.64**

**Table 6:** Transfer learning experimental results.

Dataset	Method	WT Dice	TC Dice	ET Dice
BraTS2021	Direct Migration	90.15	86.32	83.78
BraTS2021	Fine-tuning migration	91.84	88.05	85.92
TCGA-GBM	Direct Migration	85.42	80.13	76.45
TCGA-GBM	Fine-tuning migration	89.37	85.21	82.67

## Data Availability

The BraTS 2020 dataset used in this study is publicly available at https://www.med.upenn.edu/cbica/brats2020/ (accessed on 15 July 2025). The code supporting the findings of this study is available from the corresponding author upon reasonable request.

## Abbreviations

The following abbreviations are used in this manuscript:

BCE	Binary Cross Entropy
BraTS	Brain Tumor Segmentation Challenge
CNN	Convolutional Neural Network
DDM	Dense Multi-scale Module
DGA	Dense Parallel Global Attention
DSN	Dense Block
ED	Peritumoral Edema
ET	Enhancing Tumor
FLAIR	Fluid-Attenuated Inversion Recovery
GSA	Global Spatial Attention
HD	Hausdorff Distance
HGG	High-Grade Glioma
LGG	Low-Grade Glioma
MRI	Magnetic Resonance Imaging
MRM	Multimodal Feature Recombination Module
TC	Tumor Core
WT	Whole Tumor
